# Proteolysis Targeting Chimeric Molecules: Tuning Molecular Strategies for a Clinically Sound Listening

**DOI:** 10.3390/ijms23126630

**Published:** 2022-06-14

**Authors:** Federica Pedrucci, Claudia Pappalardo, Giovanni Marzaro, Nicola Ferri, Alberto Ferlin, Luca De Toni

**Affiliations:** 1 Department of Medicine, Unit of Andrology and Reproductive Medicine, University of Padova, Via Giustiniani 2, 35128 Padova, Italy; federica.pedrucci@unipd.it (F.P.); claudia.pappalardo111@gmail.com (C.P.); alberto.ferlin@unipd.it (A.F.); 2 Department of Pharmaceutical and Pharmacological Sciences, University of Padova, 35131 Padova, Italy; giovanni.marzaro@unipd.it; 3 Department of Medicine, University of Padova, 35128 Padova, Italy; nicola.ferri@unipd.it

**Keywords:** Von Hippel-Lindau, thalidomide, warhead, Lipinski rule of five, molecular glue

## Abstract

From seminal evidence in the early 2000s, the opportunity to drive the specific knockdown of a protein of interest (POI) through pharmacological entities called Proteolysis Targeting Chimeric molecules, or PROTACs, has become a possible therapeutic option with the involvement of these compounds in clinical trials for cancers and autoimmune diseases. The fulcrum of PROTACs pharmacodynamics is to favor the juxtaposition between an E3 ligase activity and the POI, followed by the ubiquitination of the latter and its degradation by the proteasome system. In the face of an apparently modular design of these drugs, being constituted by an E3 ligase binding moiety and a POI-binding moiety connected by a linker, the final structure of an efficient PROTAC degradation enhancer often goes beyond the molecular descriptors known to influence the biological activity, specificity, and pharmacokinetics, requiring a rational improvement through appropriate molecular strategies. Starting from the description of the basic principles underlying the activity of the PROTACs to the evaluation of the strategies for the improvement of pharmacodynamics and pharmacokinetics and rational design, this review examines the molecular elements that have been shown to be effective in allowing the evolution of these compounds from interesting proof of concepts to potential aids of clinical interest.

## 1. Introduction

**Figure 1:**
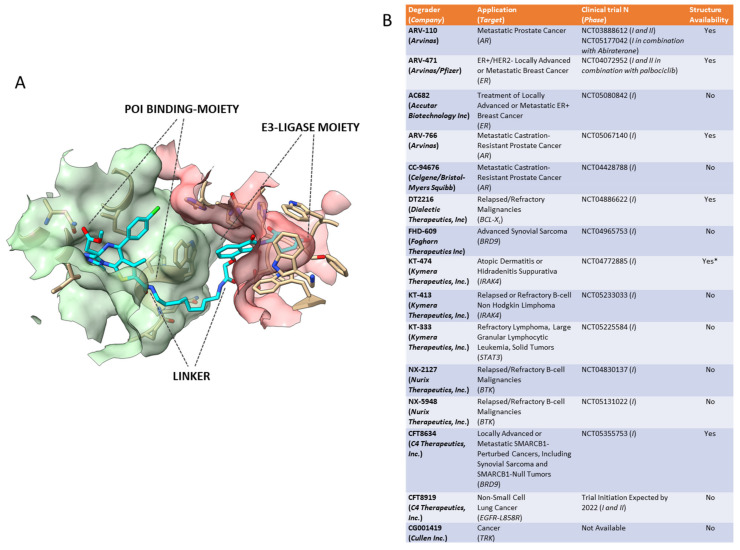
(**A**) Prototypical structure of a Proteolysis Targeting Chimeric Molecules (PROTAC) based on the example of dBET23 PROTAC (Protein Data Bank number 6BN7). PROTACs favor the formation of a ternary complex involving the PROTAC itself (cyan structure), a protein of interest (POI) in the case of the DNA damage-binding protein 1 (green surface), and a cell E3 ligase in the case of the Protein cereblon (pink surface). This is made available by the coexistence, in the same molecule, of a POI-binding moiety and an E3 ligase-binding moiety (in the case of thalidomide) joined by a linker of suitable length. (**B**) A list of PROTACS involved in clinical trials. Abbreviations: AR—androgen receptor; ER—oestrogen receptor; BCL-xL—B cell lymphoma- extra-large; BRD9—bromodomain-containing protein 9; BTK—Bruton’s tyrosine kinase; EGFR-L585R—epidermal growth factor receptor harboring the exon point mutation L858R; ER—oestrogen receptor; HS—hidradenitis suppurativa; IND-e—IND- enabling preclinical studies; IRAK4—interleukin-1 receptor-associated kinase 4; STAT3—signal transducer and activator of transcription 3; TRK—tropomyosin receptor kinase. * Ingo Hartung su Twitter: “The plot thickens regarding the structure of IRAK4 PROTAC KT-474 @KymeraTx. 2 patents disclosing deuterated, crystalline & salt forms of a single PROTAC. Comprising a non-conventional CRBN binder the chemists at Kymera seem to like a lot. More common NH-linked IMiD in KT-413? https://t.co/O8JMRLZvpY”/Twitter (accessed on 1 March 2022).

The paper from Sakamoto et al. in 2001 was the first original proof of the concept that cell degradation of specific a protein of interest (POI), by ubiquitin-dependent proteolysis, can be artificially triggered by a properly conceived pharmacological agent displaying simultaneously a POI-ligand and a E3-ubiquitin–protein ligase moiety in the same molecule (Figure 1A). In this case, it was the unrelated methionine aminopeptidase-2 degradation by the Skp1-Cullin-F box complex (SCF) induced by a deca-peptide of the IκBα negative regulator of NFκB, an SCF ligand, linked to ovalicin, a ligand of MetAP-2 protein [1]. The first proteolysis targeting chimeric molecule or “PROTAC” was then born. The opportunity to pharmacologically reprogram the E3 ligases activity towards a specific POI was rapidly tapped to knock down disease-causing proteins but, differently from a genetic method, with properly conceived small-molecule agents. In the last two decades, there has been a real flourishing of new molecular approaches aimed at improving the specificity, efficacy, safety, and pharmacokinetic profile of this class of compounds (Reviewed in [2]). In 2019, the first PROTAC drug ARV-110 entered a clinical trial for the treatment of prostate cancer, and only 3 years later, there are at least fifteen clinical trials aimed to evaluate the clinical efficacy of as many new PROTACs in the context of neoplastic and chronic-degenerative pathologies [3,4]. (Figure 1B).

This review aims to summarize which molecular approaches proved to be successful in developing a new protein degradation enhancer involved in a clinical trial.

### 1.1. The Essential: The Formation of the Ternary Complex E3 Ligase–PROTAC–POI

The fulcrum of the PROTAC’s pharmacodynamics is to allow the juxtaposition between E3 ligase and the POI through the formation of a sufficiently stable ternary complex, within which the ubiquitination of the POI can take place. Ubiquitinated POI will then be delivered to the proteasome for degradation [5]. The E3 ligase-mediated ubiquitination is actually the last in a multistep process involving the ATP-dependent activation of ubiquitin, operated by E1 enzymes, in the transfer of activated ubiquitin to a specific Cys residue of one member of the ubiquitin-conjugating E2 enzyme family and the donation of conjugated-ubiquitin to protein substrates through the ubiquitin–protein ligase E3 complexes [6]. Among the over 600 members of the E3 family, only a few can be recruited according to the availability of recognized specific ligands. These are the ligands of cereblon (CRBN), such as thalidomide, pomalidomide, and lenalidomide [7], the ligands of the cellular inhibitor of apoptosis protein 1 (cIAP1), such as bestatin [8], the ligands of mouse double-minute 2 homolog (MDM2), such as nutlin [9], and ligands of Von Hippel-Lindau (VHL) [10].

**Figure 2:**
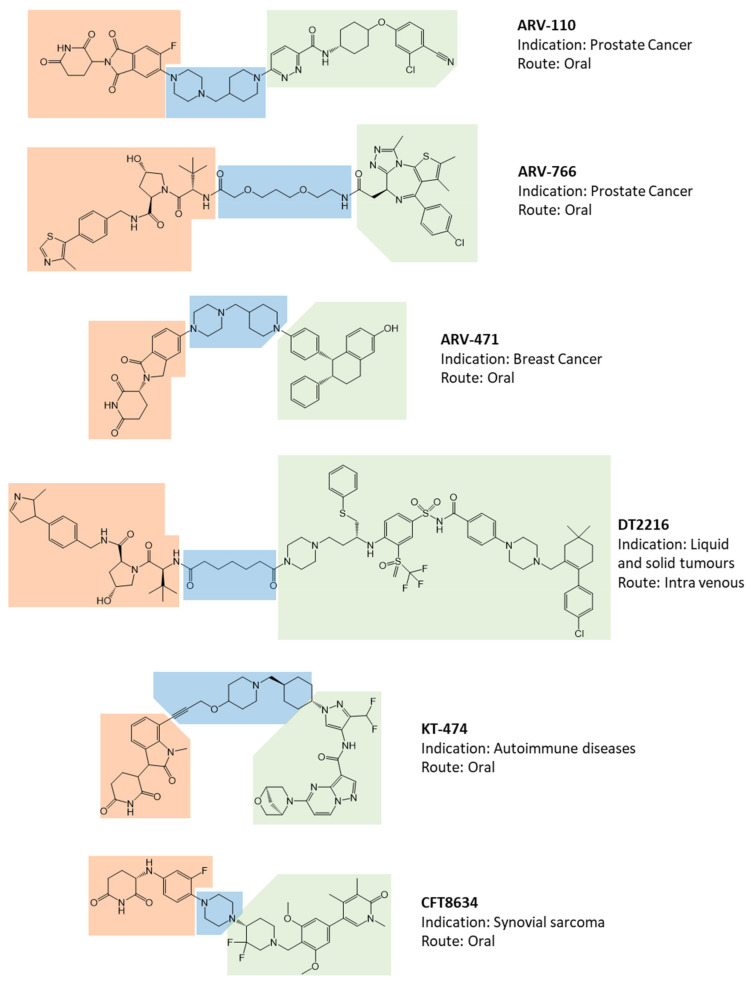
Available chemical structures, identification codes, indication, and route of administration of Proteolysis Targeting Chimeric molecules (PROTACs) involved in clinical trials. The molecular portions underlined in orange, green, and blue represent the E3 ligase-binding moiety, the protein of interest-binding moiety, and the linker, respectively.

To date, of the fifteen compounds included in clinical trials, the only two recognized VHL-based degradation enhancers are ARV-766 (from Arvinas, New Haven, CT, USA) and DT2216 (from Dialectic Therapeutics, Dallas, TX, USA, Figure 2). In contrast, CRBN-based PROTACs appear as the first-choice option in making a compound of clinical relevance. This is essentially attributable to pharmacokinetic reasons since thalidomide and derivatives, such as E3 ligase-binding moiety, show lower molecular weight and favorable hydrogen bond donors/acceptors balance and CLogP [11] (see below). In this regard, working on available pharmacokinetic cassette studies on PROTACs and conventional small molecules in animal models, Pike et al. showed that PROTACs derived from the prototypical parent ligands thalidomide, pomalidomide, and lenalidomide, display an oral bioavailability greater than 30% compared with other homologous molecules [12]. In agreement with this model, the VHL-based PROTAC DT2216 requires intravenous administration, unlike most of the other compounds under evaluation [4]. Interestingly, despite the fact that the molecule CFT8634 (C4 Therapeutics, Watertown, MA, USA) is claimed as a CRBN-based PROTAC, the molecule’s structure shows no thalidomide derivatives other than a residual glutarimide structure, probably representing the result of a deep rationalization in drug design, involving the essential moiety involved in interaction with the E3 ligase [13].

The expansion of the panel of E3 ligases or factors involved in protein degradation is currently the focus of a growing scientific interest aimed at improving key elements such as tissue or cell specificity.

The cooperative interaction among E3 ligase, PROTAC, and POI, aimed to favor the formation of a ternary complex, is proved to be associated with the POI’s degradation efficiency [14]. However, on the basis of this simple principle alone, the degradation efficiency of a PROTAC is not linearly related to the compound’s concentration; the likelihood of unproductive binary complexes forming, such as E3 ligase–PROTAC and PROTAC–POI, increases along with the PROTAC’s concentration, resulting in a bell-shaped concentration-effect curve, also known as “hook effect” [15]. On the other hand, the occurrence of the hook effect is less probable as the cooperative interaction between E3 ligase and POI increases within the ternary complex [16]. In parametric terms, given a Kd_binary_ for the binary complex between PROTAC and POI, and a Kd_ternary_ for the ternary complex E3 ligase–PROTAC–POI, a cooperative interaction exists when Kd_binary_ > Kd_ternary_. Defining the cooperativity factor α = Kd_binary_/Kd_ternary_, the positive contribution of protein–protein interactions (PPI) towards the formation of the ternary complex is provided for α > 1, while an impediment is provided for α < 1 [16,17]. The consistency of this model has been investigated in the bromodomain and extraterminal (BET) protein family involved in the epigenetic regulation of gene expression through the lysine residues acetylation of histones [18]. VHL-based PROTACs developed to knockdown BRD4, a recognized BET protein involved in malignant tumors [19], were able to favor a cooperative interaction between the E3 ligase and POI at different lines of investigation, form a large protein–protein interaction surface identified by X-ray crystallography to the direct quantification of Kd_binary_ and Kd_ternary_ by isothermal calorimetry titration (ITC) [16,20]. Furthermore, data from the degradation of the BET protein BRD4^BD2^ by the VHL-based PROTAC MZ1 let us hypothesize that the rate-limiting step of BRD4^BD2^ degradation is the ubiquitination by the E3 ligase. In fact, the degradation efficiency of the BET protein was proportional to the half-life of the ternary complex evaluated by surface plasmon resonance (SPR) [21]. This evidence largely sustains the role of the stability of the ternary complex on the degradation efficiency.

On these bases, low levels of affinity of the PROTAC for the POI can be largely compensated by the positive cooperativity for a ternary complex with the E3 ligase, as recently shown for PROTAC aimed to degrade p38α mitogen-activated protein kinase or the androgen receptor (AR) [22,23]. This aspect is of particular interest for those POIs considered to be undruggable for the lack of suitable binding sites for small molecules, but that can equally be targeted by PROTACs of lower binding affinities [14]. Thus, the POI ligand moiety, or warhead, representing the most variable part in a PROTAC’s structure through the interaction with the molecular target, is not required to necessarily have a functional role, such as an enzyme inhibitor or a receptor agonist/antagonist; it simply has to bind the POI with sufficient stability to favor the formation of a ternary complex with the E3 ligase [14]. According to this principle, it is possible to distinguish which PROTACs of clinical interest show known ligands, target known binding sites for POI, or target alternative binding sites equally functional to protein degradation.

The AR is certainly the most exploited molecular target for the treatment of prostate cancer also for PROTACs [24,25]. Importantly, the structure is available for two of the three PROTAC molecules included in clinical trials aimed at the treatment of prostate cancer, specifically ARV-110 and ARV-766 (both from Arvinas, Figure 2 [4]). The POI-binding moiety of ARV-110 displays a terminal 3-chloro-4 nitril-phenyl-terminal structure, which is frequently shared by other selective AR modulators and targets the classical ligand-binding domain (LDB) of the receptor [26,27]. In contrast, the POI-binding moiety of ARV-766 shows an unprecedented tricyclic structure with condensed rings. However, Nagata et al. recently provided an interesting structure–activity analysis and validation of novel tricyclic tetrahydroquinolines as binding moieties of the AR-LBD [28]. Accordingly, based on the available data, all AR-targeting PROTACs share the “classical” LBD of protein as the interaction interface with the warhead. Overlapping considerations can be made for ARV-471 (from Arvinas), the only oestrogen receptor-α (ER-α) targeting PROTAC for which the structure is known Figure 2 [4]. ARV-471 is currently involved in a clinical trial for the treatment of breast cancer. The POI-binding moiety of this PROTAC is actually lasofoxifene, a recognized selective estrogen receptor modulator known to bind the ER-α-LDB [29]. A separate discussion can be made for DT2216 (from Dialectic Therapeutix), representing somehow an evolution of the parent compound ABT263 (Navitoclax), a potent inhibitor of the BCL-2 anti-apoptotic protein [30]. The inhibition of BCL-2 family proteins was previously considered a promising approach for the treatment of cancer disease [31]. However, ABT263 has never found a clinical application because of the development of thrombocytopenia associated with BCL-xL inhibition [32,33]. The straightforward embedding of ABT263 in a VHL-based PROTAC was hypothesized to be effective in BCL-xL-dependent T cell acute lymphoblastic leukemia and T cell lymphoma with minor exploitation of thrombocytopenia, with E3 ligase minimally expressed in platelets [34,35].

With the exception of these few molecules, there is a general lack of data for the remaining PROTACs’ clinical relevance. In this regard, the only information about the structure of the CRBN-based PROTAC KT-474 (from Kymera/Sanofi, Kymera Therapeutics, Watertown, MA, U.S.A. and Sanofi Aventis, Bridgewater, NJ, USA.) targeting the interleukin-1 receptor-associated kinase 4 (IRAK4) derives from unofficial sources (Figure 2). IRAK4 is involved in innate immunity signaling, activated by toll-like receptors and interleukin 1-receptor IL-1Rs, through the formation of a multiprotein complex called myddosome [36]. The role of IRAK4 in the assembly of the myddosome relies on both its kinase activity and its scaffolding function, the former involved in the downstream events of the IRAK4–JNK axis, the latter involved in NF-κB activation [37]. On this base, the development of an IRAK4 degradation enhancer was thought to allow the simultaneous blocking of both kinase activity and the scaffolding functions [38]. The putative POI-binding moiety of KT-474 is an unprecedented record in the literature. However, the saturated hetero-bicycle heptane terminal and the fluoromethyl-pyrazole of the warhead resemble the structure of imatinib, a recognized inhibitor of the IRAK4-kinase activity [39]. Thus, the major hypothesis is that KT-474 relies on a novel binding moiety targeting the known kinase domain of the POI.

Finally, CFT8634 (from C4 Therapeutics) probably represents the prototype of a PROTAC by definition (Figure 2). It is a CRBM-based degradation enhancer of the bromodomain-containing protein 9 (BRD9), a subunit of the mammalian switch sucrose nonfermentable (mSWI/SNF) enzyme family of the ATP-dependent chromatin remodeling complexes, regulating the chromatin DNA accessibility and the appropriate control of gene expression [40]. The histone acylated-lysine reading activity of BRD9 has drawn attention as a possible drug target because of its specific role in synovial sarcoma and acute myeloid leukemia [41]. However, BRD9 inhibitors showed nonselective antiproliferative effects unless being part of larger molecules containing the thalidomide-like moiety [42]. Accordingly, CFT8634, whose warhead is the result of rationalization of previous BRD9 binders [43], represents the first PROTAC entering a clinical trial through the target of a novel POI for which the warhead, taken as a stand-alone small inhibitory molecule, proved pharmacologically ineffective.

### 1.2. The Step Forward: Improvement of PROTACs Specificity and Pharmacodynamics

As such, the classic PROTACs suffer from some problems common to other drug classes, such as an amenable specificity, and other more peculiar issues such as the limited cell permeability due to the large sizes. To solve these issues, several molecular approaches have been, and currently are, under investigation.

In order to reduce cell toxicity as the off-target effect caused by PROTACs, various strategies allowing the localized activation of these drugs have been devised. The spatiotemporal regulation of PROTAC’s activity can be obtained by the use of photocage or photo-switch technologies aimed at creating molecules with light-dependent activation [44]. This is the case of photocaged PROTACs (pc-PROTACs), drugs owning photocaged groups that, on the one hand, prevent the formation of the ternary complex and, on the other hand, can be removed by the irradiation with light of a suitable wavelength. These PROTACs are thus rapidly activated to degrade POIs upon irradiation with UV light [44]. In this regard, Xue et al. designed a pc-PROTAC, called pc-PROTAC1, which aimed to enhance the degradation of the BET protein BRD4 through the release of dBET1 upon irradiation with light at 365 nm of wavelength [45]. The design of pc-PROTAC1 started from a CRBN-based PROTAC and used 4,5-Dimethoxy-2-nitrobenzyl (DMNB) as a photo-caging group placed in the linker moiety. In the same study, the authors provided proof of the efficiency of this approach for another pc-PROTAC, the pc-PROTAC3. This is another CRBN-based PROTAC drug designed to enhance the protein degradation of the Bruton’s tyrosine kinase (BTK). BTK plays a crucial role in B cell development and is currently a suitable drug target for the treatment of B cell malignancies. Owning a photo-caging DMNB group on the thalidomide moiety, pc-PROTAC3 showed an exclusive light-dependent degradation of BTK [45]. Despite the wide availability of photo-caging groups and the recognized exclusive photo-activated degradation enhancement, there is no clear evidence of a greater degradation efficacy of pc-PROTACs compared with traditional PROTACs [46,47,48,49]. One of the major issues of the described technology is the irreversible activation of PROTACs upon light irradiation, with the process of protein degradation ending upon the drug’s clearance. As a possible solution, various groups have developed PROTAC molecules combined with photo-switches that can be reversibly activated and inactivated by light exposure [44]. Examples of this approach are photo-PROTACs, molecules featured by photo-switchable activation by the insertion of an azobenzene group in the linker structure. The basic principle of these drugs is that the linker is required to have a minimum length to allow the binding between the POI and the E3 ligase. Different lengths of the same linker moiety are obtained by the cis-trans geometry interconversion of an azobenzene structure, resulting in an inactive or active PROTAC, respectively. In this regard, Pfaff et al. [48] designed a highly stable and inactive cis-photo-PROTAC that, upon light irradiation at 415 nm, isomerizes into trans-photo-PROTAC displaying the correct length to allow the formation of the ternary complex and the subsequent degradation of the POI. When necessary, the drug can be “switched off” again by irradiation with a second light pulse at 530 nm that isomerizes back the molecule to the cis- form.

Despite promising light activation of photo-switchable PROTACs, this has not led to drugs involved in clinical trials so far. Most importantly, a key point of this approach is the availability of devices for tissue-specific light delivery. In fact, light supplied by classical sources, such as lamps or laser optical devices, is largely absorbed or scattered by tissue constituents, resulting in major limitations for the treatment of diseases located on the skin, underneath the skin, or on the surfaces of organs exposed outwardly through open surgery [50]. Major advances have been achieved with the development of cancer phototherapy, which is based on the intr-atumoral generation of reactive oxygen species (ROS) through the local administration of light of a proper wavelength, and photosensitizers involved in ROS production [50]. Flexible and deformable optical light-emitting diodes (LEDs) have been developed, embedded into ultrathin sheets of elastomer, and shaped to provide a planar light source. The flexibility and stretchability of these devices allow intimate contact with skin or organs in larger areas [51]. Even more interestingly, injectable optical LEDs devices placed on the catheter or needle-like carriers have been created to deliver the light source within deep tissues or organs, allowing minimal invasiveness and the possibility of accessing more internal sites [52].

However, these strategies are not exempt from some issues, such as the highly integrated assembly of nano/micro-sized electronic devices and the dissipation of the heat generated by concentrated electronics [50]. The improvement of these aspects will represent the keystone for the clinical application of this novel branch of phototherapy.

In addition to localized activation, another issue of PROTACs to be improved is a large size, representing a limitation for cell permeability. Lebraud et al. have devised a system that allows the formation of PROTACs directly inside the cells from two smaller and more permeable molecules [53]. Specifically, the authors used a CLick-formed Proteolysis TArgeting Chimera, or CLIPTAC, approach to degradation of BRD4, using a tetrazine-labeled thalidomide derivative (Tz-thalidomide) and an inhibitor of the POI, JQ1, tagged with *trans*-cyclo-octene (JQ1-TCO). The two precursors were demonstrated to assemble intracellularly and enhance ubiquitination and elimination of the protein BRD4 with the involvement of the E3 ligase CRBN. The same methodology was used by the authors to develop a further CLIPTAC aimed at enhancing the degradation of ERK1/2 [53]. The CLIPTACs self-assembled outside of cells was experimentally confirmed not to be associated with the degradation of POI, supporting the role of technology as a solution to the low permeability caused by the large molecular weight of these drugs.

A new generation of PROTACs has recently been developed using the antibody drug-conjugated (ADC) technology for targeting the delivery of the molecule into tumor target cells. This is made possible through the binding of PROTAC with a monoclonal antibody that recognizes a specific receptor present on the surface of cancer cells but not of normal cells, such as HER2 [14]. An example of PROTACs generated by the ADC approach is Ab-PROTAC3, a VHL-based trastuzumab–PROTAC conjugate that specifically recognizes HER2+ breast cancer cells and enables the degradation of the protein BRD4 in these specific cells [54]. The binding between trastuzumab and PROTAC occurs at the level of the free hydroxyl group on the VHL ligand. The link at this site, which is essential for VHL binding, leads to PROTAC activation only after the separation of the antibody during lysosomal digestion and the intracellular release of the drug.

### 1.3. More Than a Bridge between Two Shoulders: The Pharmacokinetic Role of the Linker

The role of the linker is often underestimated, but it has a critical effect on the physicochemical properties and biological activity of PROTAC compounds. Accordingly, the proper combination of ligands and linkers’ length influences the polarity and the flexibility of the molecule, setting the basis for the design of an effective PROTAC [55]. However, because of their generally high molecular weight, PROTACs suffer from major trans-murality issues with obvious implications for their pharmacokinetic profile. Possible molecular descriptors associated with drug activity or pharmacokinetics have been summarized by Lipinski et al. in “the rule of 5”, according to which a poor absorption or trans-membrane diffusion is estimated when the molecule displays more than 5 hydrogen bond donors, 10 hydrogen bond acceptors, a molecular weight greater than 500, and a calculated octanol/water partition coefficient (CLogP) greater than 5, or its Moriguchi variant (MlogP) greater than 4.15 [56]. Additionally, Veber et al. suggested possible criteria for the estimation of a good oral bioavailability in rodent models for molecules showing 10 or fewer rotatable bonds and a polar surface area equal to or less than 140 Å^2^, or ≤12 H-bond donors/acceptors [57]. In general, the molecular weight of known PROTACs ranges from 900 to 1100 Da, and the number of rotatable bonds stands between 20 and 25 [11,58]. Thus, despite a recognized efficacy as a protein degradation enhancer, a PROTAC may be prevented from achieving its molecular targets by the poor pharmacokinetic profile because of low oral absorption and cell permeability. Since the two ligand moieties are considered almost invariant in the design of a PROTAC, the linker remains practically the only “editable” structure deeply influencing the physicochemical properties of the overall molecules. Normally, a PROTAC linker has three different moieties: an E3 ligase-binding part, a body of various compositions and lengths, and a POI-ligand-binding part. The distance between the two moieties optimal for promoting efficient ubiquitination needs to be determined on a case-by-case basis. Previous studies have shown that linker length may be an important discriminating factor for PROTAC activity [59]. If the linker is too short, the two ligands cannot simultaneously bind to their respective proteins due to steric clashes resulting in the failure of ternary complex formation. On the other hand, if the linker is too long, E3 ligase cannot be brought in close proximity to the POI for its ubiquitination. 

Currently, structural optimization of PROTACs mainly focuses on the evaluation of the structure–activity relationship (SAR) for various size linkers [55]. Previous studies have also demonstrated that the linker’s length does not influence the target binding affinity but can instead have a major impact on cytotoxicity since longer linkers have been shown to be more toxic [60]. Conversely, PROTACs with longer linkers show increased efficiency in mediating target protein degradation during the ubiquitination process. As a result, there is no general linker-design strategy that has been planned so far. The most common motifs incorporated into PROTAC linker structures are poly-ethylene glycol (PEG) units and alkyl chains of varying lengths, representing nearly 55% and 30% of available linkers, respectively. Around 65% of structures contain both alkyl and PEG segments, whilst a further 15% used modifications of the individual glycol units, incorporating additional methylene moieties to access different chain lengths. Other represented motifs include alkynes (7%), triazoles (6%), and saturated heterocycles such as piperazine and piperidine (4% each) [61]. In this regard, the two protein degradation enhancers ARV-110 and ARV-471, involved in clinical trials, display piperidine and piperazine-based linker with lower conformational freedom compared with their parent molecules of similar length. In addition, KT-474 and CFT8634 degraders show mixed piperidine and piperazine-based linkers. As previously cited, alkyl or rigid linkers differ from the PEG-based subsets by having a higher cLogP, lower topological polar surface area (TPSA), and fewer hydrogen bond acceptors, likely supporting cell permeability and favorable pharmacokinetics [62] (Figure 2).

Once these general considerations were clarified, an interesting insight into the pharmacokinetic role of linkers from a physical–chemical perspective was recently provided in a study by Atilaw et al., where authors aimed to interpret the biological activity of PROTAC 1, a VHL-based degradation enhancer of the e potential cancer target extracellular signal-regulated kinase 5 [62,63]. The comparison among the calculated molecular descriptors for PROTAC 1 and those of a panel of 217 VHL-based PROTACs from Maple et al. [58] showed an apparent inconsistency with biological activity since PROTAC 1, despite being placed beyond the threshold value for Lipinski’s and Veber’s rules, displayed medium to high cell permeability. Interestingly, this evidence was much more interpretable in the light of the spectrum of conformations assumed by PROTAC 1 in an apolar environment, derived from an integrated approach using the NMR analysis of molecular flexibility in solution (NAMFIS) algorithm [64,65]. In particular, the apolar environment generated by the chloroform solvent, compared with more polar solvents such as water–dimethyl sulfoxide mixtures, emphasized folded conformations of PROTAC 1stabilized by intramolecular interactions such as intramolecular hydrogen bonds, largely associated with PEG-based linkers, and π−π interactions, resulting in low values of the molecular descriptor solvent-accessible 3D polar surface area (SA 3D PSA) [66]. Since the center of a cell membrane owns a polarity closed to that of chloroform, it can be hypothesized that the compact configuration assumed by PROTAC 1 in this environment is more compatible with satisfactory passive cell permeability [62]. Despite ancillary, this evidence supports a role for flexible, PEG-based linkers in conferring a chameleonicity to this type of PROTACs, with a major impact on cell permeability. It is interesting to note that the degradation enhancer ARV-766, currently involved in a trial for prostate cancer, owns a molecular weight greater than 500 Da, a PEG-based linker, and a VHL E3 ligase moiety but contrary to what could be expected based on the rule of five is registered as an oral drug [4]. It is, therefore, conceivable that for this molecule, there is a high degree of chameleonicity with favorable consequences on cell permeability [12] (Figure 2). It can therefore be deduced that proper rationalization of the linker structure represents the likely gap to be filled in order to convert a PROTAC conceived as a proof of concept to evaluate the possible efficacy of the drug in preclinical studies to a clinical candidate with an improved pharmacokinetic profile.

### 1.4. Current Approaches for a Rational Design of PROTACs

The PROTAC acts through a complex dynamic process, which implies the recognition by the unbound PROTAC at one side of the first protein (either the E3 ligase or the POI), the recognition by the liganded PROTAC at the other side of the second protein (either the POI or the E3 ligase), and finally, the rearrangement of the supramolecular complex to the effective ternary complex [1]. Accordingly, the PROTAC linker must not only possess the right length (so that the small molecule can interact with both proteins at the same time) but also proper flexibility. Indeed, during the last step, the PROTAC linker must collapse to allow the proteins to get in close contact. The features of the linker were clearly demonstrated in 2017 by Gadd et al., who resolved for the first time the crystallographic structure of a ternary complex [16].

Such a highly dynamic mechanism of action is very difficult to investigate with standard computational tools commonly used for virtual screening purposes. Moreover, the degrees of freedom that intrinsically characterize the linker generate an entropic contribution to the binding that is very hard to account for with molecular docking tools without applying specific methods of correction [66]. Accordingly, the most common design strategy for PROTACs relies on “try and test” procedures.

Nevertheless, some research groups are actively focusing on computational methodologies to accelerate the rational discovery of new PROTACs. Since 2017, several other ternary complexes have been resolved, paving the way for the structure-based design of PROTACs and even for the discovery of more rigid PROTACs with improved potency and selectivity [4,67].

A set of mathematical tools that describe the thermodynamics and kinetics of ternary complex formations starting from biochemical data has been reported very recently [68]. These tools aim at deriving structure–activity relationships (SARs) data that are in turn used to empirically design improved PROTACs. Guo et al. applied molecular dynamics simulations to investigate the effect of different PROTACs on the stability of the ternary complexes. The study demonstrated higher dynamic stability of the ternary complex in the case a covalent bond was formed with the PROTAC [69]. Another interesting approach has been reported by Dummond et al., who implemented four methods in the MOE docking suite to virtually screen PROTACs. The methodology has been further improved by adding an additional clustering step [20,69,70]. Even more recently, a new tool named PRosettaC has been introduced that was able to reproduce the structure of the known ternary complexes by alternating protein–protein docking and protein–ligand docking procedures [71]. The tool finally aims at supporting the rational design of novel PROTACs, although, in the paper, no new compounds were reported. The tool is currently available through a webserver (https://prosettac.weizmann.ac.il/ (accessed on 1 February 2022)). Finally, in 2021 Bai et al. reported a protocol for PROTAC design through molecular docking based on the Rosetta engine [72]. The method involves different steps. First, a protein–protein docking is conducted, with both E3 ligase and POI bound to the respective small molecules (i.e., the two heads of the final PROTAC). Then, a series of low-energy linker conformers are generated; these conformers are then aligned on the putative protein complexes generated in the first step. If a linker is able to connect the ligand moieties, a potential PROTAC is generated by merging the structures and docked against the protein complex to confirm its ability to form a ternary complex. 

## 2. Conclusions and Future Perspectives

**Figure 3:**
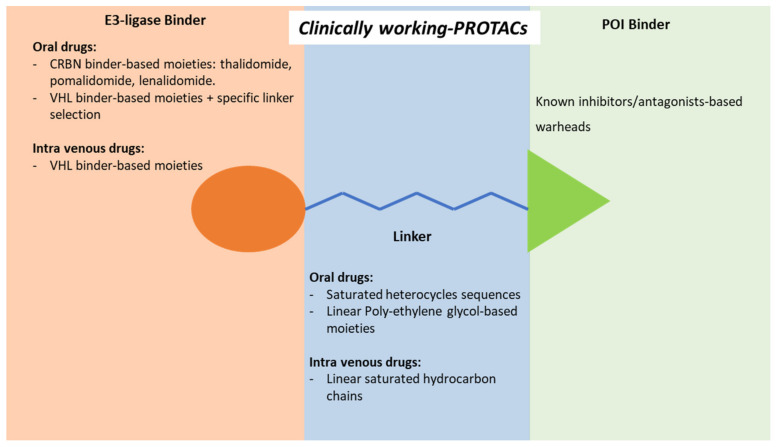
Structural characteristics of Proteolysis Targeting Chimeric molecules (PROTACs) currently in a clinical trial, distinguished by structural moiety and route of administration.

During two decades, PROTACs evolved from the proof of concept of the derived degradation of a specific protein of interest to an arsenal of molecules entered in clinical trials for the treatment of cancer and chronic degenerative diseases. The principles of PROTACs pharmacodynamics rely on a three-moieties molecule: an E3 ligase-binding moiety on the one hand, a POI-binding moiety on the other hand, and a linker connecting each other. Importantly, in principle, the POI-binding moiety is not required to have a functional role, such as an inhibitor or an antagonist, but should simply bind the targeted protein with sufficient stability to favor the formation of a ternary complex in which ubiquitination can occur [14]. Despite the number of molecular approaches developed to improve the degradation efficiency of PROTAC molecules, the structure analysis of compounds with such a clinical efficacy to allow their inclusion in a clinical trial translates into a few stringent “tips” for the design of a potentially successful drug, summarized in Figure 3. In detail:

- The opportunity to obtain an oral drug essentially relies on the use of a CRBN-based degradation enhancer. In fact, the corresponding thalidomide-like moiety displays a favorable lipophilic/molecular weight ratio and consequent satisfactory gut absorption. Unless a deep design intervention on the linker, a VHL-based degradation enhancer, the only other alternative to the CRBN, is essentially suitable for parenteral administration;
- According to available data, with the sole exception of BRD9 degradation enhancer CFT8634, all PROTAC’s warheads of clinical interest target the known POI’s binding sites through recognized ligands or structural derivatives. Data on the use of alternative binding sites of known target proteins are not available;
- Despite the simple presumptive role of the linker portion, it represents a structural moiety of design interest. The use of saturated heterocycles or on available linkers with hydrogen bond acceptors and reduced conformational freedom is frequently associated with the development of oral drugs. Saturated linear hydrocarbon chains are more frequently found in parenteral administration.

In summary, current, new generations of molecular glue degraders, in contrast to PROTACs, are likely to display pharmacokinetic features of small molecules, representing a promising evolution of PROTAC as anticancer therapy.

## Data Availability

Supporting data and repository are freely available at the links provided within the text.

## References

[1] Sakamoto K.M., Kim K.B., Kumagai A., Mercurio F., Crews C.M., Deshaies R.J. (2001). Protacs: Chimeric Molecules That Target Proteins to the Skp1-Cullin-F Box Complex for Ubiquitination and Degradation. Proc. Natl. Acad. Sci. USA.

[2] Hughes S.J., Testa A., Thompson N., Churcher I. (2021). The Rise and Rise of Protein Degradation: Opportunities and Challenges Ahead. Drug Discov. Today.

[3] Mullard A. (2019). First Targeted Protein Degrader Hits the Clinic. Nat. Rev. Drug Discov..

[4] Békés M., Langley D.R., Crews C.M. (2022). PROTAC Targeted Protein Degraders: The Past Is Prologue. Nat. Rev. Drug Discov..

[5] Amm I., Sommer T., Wolf D.H. (2014). Protein Quality Control and Elimination of Protein Waste: The Role of the Ubiquitin–Proteasome System. Biochim. Biophys. Acta Mol. Cell Res..

[6] Hershko A., Ciechanover A. (1998). The Ubiquitin System. Annu. Rev. Biochem..

[7] Ito T., Ando H., Suzuki T., Ogura T., Hotta K., Imamura Y., Yamaguchi Y., Handa H. (2010). Identification of a Primary Target of Thalidomide Teratogenicity. Science.

[8] Itoh Y., Ishikawa M., Naito M., Hashimoto Y. (2010). Protein Knockdown Using Methyl Bestatin–Ligand Hybrid Molecules: Design and Synthesis of Inducers of Ubiquitination-Mediated Degradation of Cellular Retinoic Acid-Binding Proteins. J. Am. Chem. Soc..

[9] Logan I.R., McNeill H.V., Cook S., Lu X., Lunec J., Robson C.N. (2007). Analysis of the MDM2 Antagonist Nutlin-3 in Human Prostate Cancer Cells. Prostate.

[10] Zengerle M., Chan K.-H., Ciulli A. (2015). Selective Small Molecule Induced Degradation of the BET Bromodomain Protein BRD4. ACS Chem. Biol..

[11] Edmondson S.D., Yang B., Fallan C. (2019). Proteolysis Targeting Chimeras (PROTACs)in ‘beyond Rule-of-Five’ Chemical Space: Recent Progress and Future Challenges. Bioorg. Med. Chem. Lett..

[12] Pike A., Williamson B., Harlfinger S., Martin S., McGinnity D.F. (2020). Optimising Proteolysis-Targeting Chimeras (PROTACs) for Oral Drug Delivery: A Drug Metabolism and Pharmacokinetics Perspective. Drug Discov. Today.

[13] Mori T., Ito T., Liu S., Ando H., Sakamoto S., Yamaguchi Y., Tokunaga E., Shibata N., Handa H., Hakoshima T. (2018). Structural Basis of Thalidomide Enantiomer Binding to Cereblon. Sci. Rep..

[14] Hu Z., Crews C.M. (2022). Recent Developments in PROTAC-Mediated Protein Degradation: From Bench to Clinic. ChemBioChem.

[15] Pettersson M., Crews C.M. (2019). PROteolysis TArgeting Chimeras (PROTACs)—Past, Present and Future. Drug Discov. Today Technol..

[16] Gadd M.S., Testa A., Lucas X., Chan K.H., Chen W., Lamont D.J., Zengerle M., Ciulli A. (2017). Structural Basis of PROTAC Cooperative Recognition for Selective Protein Degradation. Nat. Chem. Biol..

[17] Zhang Y., Loh C., Chen J., Mainolfi N. (2019). Targeted Protein Degradation Mechanisms. Drug Discov. Today Technol..

[18] Stathis A., Bertoni F. (2018). BET Proteins as Targets for Anticancer Treatment. Cancer Discov..

[19] Chen L., Liu Z., Li X. (2022). Recent Advances in Dual BRD4-Kinase Inhibitors Based on Polypharmacology. ChemMedChem.

[20] Testa A., Hughes S.J., Lucas X., Wright J.E., Ciulli A. (2020). Structure-Based Design of a Macrocyclic PROTAC. Angew. Chem..

[21] Roy M.J., Winkler S., Hughes S.J., Whitworth C., Galant M., Farnaby W., Rumpel K., Ciulli A. (2019). SPR-Measured Dissociation Kinetics of PROTAC Ternary Complexes Influence Target Degradation Rate. ACS Chem. Biol..

[22] Bondeson D.P., Smith B.E., Burslem G.M., Buhimschi A.D., Hines J., Jaime-Figueroa S., Wang J., Hamman B.D., Ishchenko A., Crews C.M. (2018). Lessons in PROTAC Design from Selective Degradation with a Promiscuous Warhead. Cell Chem. Biol..

[23] Han X., Zhao L., Xiang W., Qin C., Miao B., Xu T., Wang M., Yang C.Y., Chinnaswamy K., Stuckey J. (2019). Discovery of Highly Potent and Efficient PROTAC Degraders of Androgen Receptor (AR) by Employing Weak Binding Affinity VHL E3 Ligase Ligands. J. Med. Chem..

[24] Pulliam T.L., Goli P., Awad D., Lin C., Wilkenfeld S.R., Frigo D.E. (2022). Regulation and Role of CAMKK2 in Prostate Cancer. Nat. Rev. Urol..

[25] Kargbo R.B. (2020). PROTAC Compounds Targeting Androgen Receptor for Cancer Therapeutics: Prostate Cancer and Kennedy’s Disease. ACS Med. Chem. Lett..

[26] Schlienger N., Lund B.W., Pawlas J., Badalassi F., Bertozzi F., Lewinsky R., Fejzic A., Thygesen M.B., Tabatabaei A., Bradley S.R. (2009). Synthesis, Structure-Activity Relationships, and Characterization of Novel Nonsteroidal and Selective Androgen Receptor Modulators. J. Med. Chem..

[27] Cattrini C., Caffo O., de Giorgi U., Mennitto A., Gennari A., Olmos D., Castro E. (2022). Apalutamide, Darolutamide and Enzalutamide for Nonmetastatic Castration-Resistant Prostate Cancer (NmCRPC): A Critical Review. Cancers.

[28] Nagata N., Miyakawa M., Amano S., Furuya K., Yamamoto N., Inoguchi K. (2011). Design and Synthesis of Tricyclic Tetrahydroquinolines as a New Series of Nonsteroidal Selective Androgen Receptor Modulators (SARMs). Bioorg. Med. Chem. Lett..

[29] Vajdos F.F., Hoth L.R., Geoghegan K.F., Simons S.P., LeMotte P.K., Danley D.E., Ammirati M.J., Pandit J. (2007). The 2.0 Å Crystal Structure of the ERα Ligand-Binding Domain Complexed with Lasofoxifene. Protein Sci..

[30] Hanahan D., Weinberg R.A. (2011). Hallmarks of Cancer: The next Generation. Cell.

[31] Ashkenazi A., Fairbrother W.J., Leverson J.D., Souers A.J. (2017). From Basic Apoptosis Discoveries to Advanced Selective BCL-2 Family Inhibitors. Nat. Rev. Drug Discov..

[32] Kaefer A., Yang J., Noertersheuser P., Mensing S., Humerickhouse R., Awni W., Xiong H. (2014). Mechanism-Based Pharmacokinetic/Pharmacodynamic Meta-Analysis of Navitoclax (ABT-263) Induced Thrombocytopenia. Cancer Chemother. Pharmacol..

[33] Schoenwaelder S.M., Jarman K.E., Gardiner E.E., Hua M., Qiao J., White M.J., Josefsson E.C., Alwis I., Ono A., Willcox A. (2011). Bcl-XL-Inhibitory BH3 Mimetics Can Induce a Transient Thrombocytopathy That Undermines the Hemostatic Function of Platelets. Blood.

[34] Khan S., Zhang X., Lv D., Zhang Q., He Y., Zhang P., Liu X., Thummuri D., Yuan Y., Wiegand J.S. (2019). A Selective BCL-XL PROTAC Degrader Achieves Safe and Potent Antitumor Activity. Nat. Med..

[35] Lv D., Pal P., Liu X., Jia Y., Thummuri D., Zhang P., Hu W., Pei J., Zhang Q., Zhou S. (2021). Development of a BCL-XL and BCL-2 Dual Degrader with Improved Anti-Leukemic Activity. Nat. Commun..

[36] De Nardo D., Balka K.R., Gloria Y.C., Rao V.R., Latz E., Masters S.L. (2018). Interleukin-1 Receptor-Associated Kinase 4 (IRAK4) Plays a Dual Role in Myddosome Formation and Toll-like Receptor Signaling. J. Biol. Chem..

[37] Somani V.K., Zhang D., Dodhiawala P.B., Lander V.E., Liu X., Kang L.-I., Chen H.-P., Knolhoff B.L., Li L., Grierson P.M. (2022). IRAK4 Signaling Drives Resistance to Checkpoint Immunotherapy in Pancreatic Ductal Adenocarcinoma. Gastroenterology.

[38] Chen Y., Ning Y., Bai G., Tong L., Zhang T., Zhou J., Zhang H., Xie H., Ding J., Duan W. (2021). Design, Synthesis, and Biological Evaluation of IRAK4-Targeting PROTACs. ACS Med. Chem. Lett..

[39] De Novellis D., Cacace F., Caprioli V., Wierda W.G., Mahadeo K.M., Tambaro F.P. (2021). The Tki Era in Chronic Leukemias. Pharmaceutics.

[40] Zullow H.J., Sankar A., Ingram D.R., Samé Guerra D.D., D’Avino A.R., Collings C.K., Lazcano R., Wang W.-L., Liang Y., Qi J. (2022). The FUS::DDIT3 Fusion Oncoprotein Inhibits BAF Complex Targeting and Activity in Myxoid Liposarcoma. Mol. Cell.

[41] Michel B.C., D’Avino A.R., Cassel S.H., Mashtalir N., McKenzie Z.M., McBride M.J., Valencia A.M., Zhou Q., Bocker M., Soares L.M.M. (2018). A Non-Canonical SWI/SNF Complex Is a Synthetic Lethal Target in Cancers Driven by BAF Complex Perturbation. Nat. Cell Biol..

[42] Bechter O., Schöffski P. (2020). Make Your Best BET: The Emerging Role of BET Inhibitor Treatment in Malignant Tumors. Pharmacol. Ther..

[43] Sabnis R.W. (2021). BRD9 Bifunctional Degraders for Treating Cancer. ACS Med. Chem. Lett..

[44] Liu J., Peng Y., Wei W. (2021). Light-Controllable PROTACs for Temporospatial Control of Protein Degradation. Front. Cell Dev. Biol..

[45] Xue G., Wang K., Zhou D., Zhong H., Pan Z. (2019). Light-Induced Protein Degradation with Photocaged PROTACs. J. Am. Chem. Soc..

[46] Reynders M., Matsuura B.S., Bérouti M., Simoneschi D., Marzio A., Pagano M., Trauner D. (2020). PHOTACs enable optical control of protein degradation. Sci. Adv..

[47] Jin Y.-H., Lu M.-C., Wang Y., Shan W.-X., Wang X.-Y., You Q.-D., Jiang Z.-Y. (2020). Azo-PROTAC: Novel Light-Controlled Small-Molecule Tool for Protein Knockdown. J. Med. Chem..

[48] Pfaff P., Kusal T., Samarasinghe T.G., Crews C.M., Carreira E.M. (2019). Reversible Spatiotemporal Control of Induced Protein Degradation by Bistable PhotoPROTACs. ACS Cent. Sci..

[49] Kounde C.S., Shchepinova M.M., Saunders C.N., Muelbaier M., Rackham M.D., Harling J.D., Tate E.W. (2020). A Caged E3 Ligase Ligand for PROTAC-Mediated Protein Degradation with Light. Chem. Commun..

[50] Seung Lee J., Kim J., Ye Y., Kim T. (2022). Materials and Device Design for Advanced Phototherapy Systems. Adv. Drug Deliv. Rev..

[51] Van den Brand J., de Kok M., Koetse M., Cauwe M., Verplancke R., Bossuyt F., Jablonski M., Vanfleteren J. (2015). Flexible and Stretchable Electronics for Wearable Health Devices. Solid-State Electron..

[52] Lu L., Gutruf P., Xia L., Bhatti D.L., Wang X., Vazquez-Guardado A., Ning X., Shen X., Sang T., Ma R. (2018). Wireless Optoelectronic Photometers for Monitoring Neuronal Dynamics in the Deep Brain. Proc. Natl. Acad. Sci. USA.

[53] Lebraud H., Wright D.J., Johnson C.N., Heightman T.D. (2016). Protein Degradation by In-Cell Self-Assembly of Proteolysis Targeting Chimeras. ACS Cent. Sci..

[54] Maneiro M., Forte N., Shchepinova M.M., Kounde C.S., Chudasama V., Baker J.R., Tate E.W. (2020). Antibody–PROTAC Conjugates Enable HER2-Dependent Targeted Protein Degradation of BRD4. ACS Chem. Biol..

[55] Zagidullin A., Milyukov V., Rizvanov A., Bulatov E. (2020). Novel Approaches for the Rational Design of PROTAC Linkers. Explor. Target. AntiTumor Ther..

[56] Lipinski C.A., Lombardo F., Dominy B.W., Feeney P.J. (1997). Experimental and Computational Approaches to Estimate Solubility and Permeability in Drug Discovery and Development Settings. Adv. Drug Deliv. Rev..

[57] Veber D.F., Johnson S.R., Cheng H.Y., Smith B.R., Ward K.W., Kopple K.D. (2002). Molecular Properties That Influence the Oral Bioavailability of Drug Candidates. J. Med. Chem..

[58] Maple H.J., Clayden N., Baron A., Stacey C., Felix R. (2019). Developing Degraders: Principles and Perspectives on Design and Chemical Space. MedChemComm.

[59] Cyrus K., Wehenkel M., Choi E.Y., Han H.J., Lee H., Swanson H., Kim K.B. (2011). Impact of Linker Length on the Activity of PROTACs. Mol. BioSyst..

[60] Negi A., Voisin-Chiret A.S. (2022). Strategies to Reduce the On-Target Platelet Toxicity of Bcl-XL Inhibitors: PROTACs, SNIPERs and Prodrug-Based Approaches. ChemBioChem.

[61] Troup R.I., Fallan C., Baud M.G.J. (2020). Current Strategies for the Design of PROTAC Linkers: A Critical Review. Explor. Target. AntiTumor Ther..

[62] Atilaw Y., Poongavanam V., Svensson Nilsson C., Nguyen D., Giese A., Meibom D., Erdelyi M., Kihlberg J. (2021). Solution Conformations Shed Light on PROTAC Cell Permeability. ACS Med. Chem. Lett..

[63] Pereira D.M., Rodrigues C.M.P. (2020). Targeted Avenues for Cancer Treatment: The MEK5–ERK5 Signaling Pathway. Trends Mol. Med..

[64] Danelius E., Poongavanam V., Peintner S., Wieske L.H.E., Erdélyi M., Kihlberg J. (2020). Solution Conformations Explain the Chameleonic Behaviour of Macrocyclic Drugs. Chem. Eur. J..

[65] Cicero D.O., Barbato G., Bazzo R. (1995). NMR Analysis of Molecular Flexibility in Solution: A New Method for the Study of Complex Distributions of Rapidly Exchanging Conformations. Application to a 13-Residue Peptide with an 8-Residue Loop. J. Am. Chem. Soc..

[66] Gilson M.K., Zhou H.X. (2007). Calculation of Protein-Ligand Binding Affinities. Annu. Rev. Biophys. Biomol. Struct..

[67] Han B. (2020). A Suite of Mathematical Solutions to Describe Ternary Complex Formation and Their Application to Targeted Protein Degradation by Heterobifunctional Ligands. J. Biol. Chem..

[68] Guo W.H., Qi X., Yu X., Liu Y., Chung C.I., Bai F., Lin X., Lu D., Wang L., Chen J. (2020). Enhancing Intracellular Accumulation and Target Engagement of PROTACs with Reversible Covalent Chemistry. Nat. Commun..

[69] Drummond M.L., Williams C.I. (2019). In Silico Modeling of PROTAC-Mediated Ternary Complexes: Validation and Application. J. Chem. Inf. Model..

[70] Molecular Operating Environment (MOE) (2022). 2020.09 Chemical Computing Group ULC, 1010 Sherbooke St. West, Suite #910, Montreal, QC, Canada, H3A 2R7. https://www.chemcomp.com/Products.htm.

[71] Zaidman D., Prilusky J., London N. (2020). ProsetTac: Rosetta Based Modeling of PROTAC Mediated Ternary Complexes. J. Chem. Inf. Model..

[72] Bai N., Miller S.A., Andrianov G.V., Yates M., Kirubakaran P., Karanicolas J. (2021). Rationalizing PROTAC-Mediated Ternary Complex Formation Using Rosetta. J. Chem. Inf. Model..

